# Experiencing integration: a qualitative pilot study of consumer and provider experiences of integrated primary health care in Australia

**DOI:** 10.1186/s12875-016-0575-z

**Published:** 2017-01-10

**Authors:** Michelle Banfield, Tanisha Jowsey, Anne Parkinson, Kirsty A. Douglas, Paresh Dawda

**Affiliations:** 1 Centre for Mental Health Research, Research School of Population Health, The Australian National University, 63 Eggleston Rd, Acton, ACT 2601 Australia; 2 Centre for Medical and Health Sciences Education The University of Auckland, Auckland City Hospital, Building 599, Level 12, Auckland, 1023 New Zealand; 3 Department of Health Services Research and Policy, Research School of Population Health, The Australian National University, 63 Eggleston Rd, Acton, ACT 2601 Australia; 4 ANU Medical School, Canberra Hospital, Bldg 4, Level 2, Garran, ACT 2605 Australia; 5 Ochre Health Medical Centre, Cnr Allawoona Street & Ginninderra Drive, Bruce, ACT 2617 Australia

**Keywords:** Integration, Primary health care, Consumer experience

## Abstract

**Background:**

The terms integration and integrated care describe the complex, patient-centred strategies to improve coordination of healthcare services. Frameworks exist to conceptualise these terms, but these have been developed from a professional viewpoint.

The objective of this study was to explore consumers’ and providers’ concepts, expectations and experience of integrated care. A key focus was whether frameworks developed from a professional perspective are effective models to explore people’s experiences.

**Methods:**

A qualitative pilot study was undertaken at one Australian multidisciplinary primary health care centre. Semi-structured interviews were conducted with consumers (*N* = 19) and staff (*N* = 10). Data were analysed using a framework analysis approach.

**Results:**

Consumers’ experience of integrated care tended to be implicit in their descriptions of primary healthcare experiences more broadly. Experiences related to the typologies involved clinical and functional integration, such as continuity of providers and the usefulness of shared information. Staff focused on clinical level integration, but also talked about a cultural shift that demonstrated normative, professional and functional integration.

**Conclusions:**

Existing frameworks for integration have been heavily influenced by the provider and organisational perspectives. They are useful for conceptualising integration from a professional perspective, but are less relevant for consumers’ experiences. Consumers of integrated primary health care may be more focussed on relational aspects of care and outcomes of care.

## Background

When planning and describing desired health service provision, we often find the terms ‘integration’ and ‘integrated care’ used interchangeably. However, Kodner and colleagues have pointed out that ‘integration’ refers to structures and processes, while ‘integrated care’ is more concerned with the patient experiences and outcomes of such processes [1]. Despite this distinction, confusion remains in the literature, which others have noted [1–4]. This is largely due to the complexity with which integration and integrated care engage; operating on different levels of health systems, both horizontally and vertically [1, 5]. Kodner ([6], p.12) provides a useful definition of integrated care that further demonstrates the complexity:

[a] multi-level, multi-modal, demand driven and patient-centred strategy designed to address complex and costly health needs by achieving better coordination of services across the entire care continuum. Not an end in itself, integrated care is a means of optimizing system performance and attaining quality patient outcomes.

Given there is no recognised common model of integrated care, a conceptual framework is needed to better understand integrated care and guide empirical research [2, 3, 7–10]. As Goodwin recently argued, we lack the means to effectively measure and monitor outcomes in integrated care, ‘particularly in terms of understanding improvements in the user experience’[11].

A useful starting point for demonstrating integration as a process (and for illustrating its complexity) may be the framework developed by Fulop et al. [12], which recognises the importance of process and cultural changes in addition to structures and governance. They identify six dimensions necessary for effective integration (see Table 1).

**Table 1:** Fulop et al.’s six necessary dimensions for effective integration

Necessary dimensions for effective integration^a^	Details
Organisational integration	How the organisation is formally structured
Functional integration	How the non-clinical support and back-office processes are integrated
Service integration	How the clinical services are integrated
Clinical integration	How clinical team level care pathways are organised
Normative integration	The role of shared values
Systemic integration	The coherence of policies across organisational levels.

^a^Adapted from Fulop et al. [12]

Also relevant is the taxonomy of integrated care that Valentijn and colleagues [3, 10, 13, 14] have developed, which uses a theory-driven mixed methods approach. The taxonomy, recently referred to as the Rainbow Model of Integrated Care [10] organises the six dimensions into the level of the system at which they operate (macro: systemic; meso: organisational and service/professional; and micro: clinical), with normative and functional integration described as enablers that operate across all levels. The model further identifies care as person- or population-focused and describes 21 key characteristics identified as necessary for achieving integrated care in a primary care setting (see Table 2) [3, 10, 13, 14]. However, as Valentijn and colleagues point out, not all stakeholders were engaged in their research, which consisted of Delphi studies with experts. For example, consumers were not included in the expert group, leaving the utility of these features for consumers unknown [13, 14].

**Table 2:** Key characteristics of integrated primary health care (adapted from Valentijn et al. [13])

Dimensions	Characteristics
Scope of care	
Person-focused care	1. Centrality of client needs
Population based care	2. Centrality of population needs
Dimensions and level of system	
Clinical integration (micro)	3. Case management
	4. Continuity
	5. Interaction between professional and client
	6. Individual multidisciplinary care plan
Service/Professional integration (meso)	7. Inter-professional education
	8. Agreements on interdisciplinary collaboration
	9. Value creation for the professional
Organisational integration (meso)	10. Inter-organisational governance
	11. Inter-organisational strategy
	12. Trust
System integration (macro)	13. Alignment of regulatory frameworks
	14. Environmental climate
Enablers of integration	
Functional integration	15. Learning organisations
	16. Information management
	17. Regular feedback of performance indicators
Normative integration	18. Shared vision
	19. Reliable behaviour
	20. Visionary leadership
	21. Linking cultures

The objective of the present study was to explore the perspectives of consumers and providers on integrated care within a newly-opened multidisciplinary primary healthcare centre. Of interest was how consumers with chronic illness and health care providers conceptualise integration, what they expect in terms of integrated care and what they experience. A key focus was whether the Fulop et al. model [12] and/or the Valentijn et al. taxonomy of features [13] could be effective modes through which people’s perspectives may be explored and analysed.

## Methods

The ethical aspects of the research were approved by The Australian National University Science and Medical Delegated Ethics Review Committee (protocol number 2014/651). All participants provided written consent to participate in the study and for interviews to be audio-recorded.

The research was developed and conducted according to a flexible participatory research model [15]. The research team included health professionals, consumer leaders and researchers from various disciplinary backgrounds. The team worked closely with a reference group throughout the project, comprising two consumer representatives, a General Practitioner (GP), an allied health professional, a nurse and a senior manager with the provider organisation. The reference group provided feedback on the research protocols, facilitated data collection and contributed to analysis and reporting of results.

The current study was part of a pilot project to explore methods of investigating integrated primary healthcare, including through clinical records (see McRae et al. [16]), a patient experience survey (unpublished data) and testing the utility of professionally-developed frameworks for consumer and provider experiences of integrated care (current study). As a pilot, participant numbers and the setting were purposely limited. Findings from the pilot study will be used to inform the design of broader-ranging studies of integrated primary healthcare, including different models and locations.

Data collection was conducted at a large, urban, multidisciplinary primary healthcare centre in Australia between April and August 2015. The centre had been in operation for approximately 15 months and had been funded under an Australian government program (GP Super Clinics) widely publicised in the media that aimed to promote co-location and integration of general practice with allied health and other service providers in order to more effectively support those with, or at risk of, chronic disease. The GP Super Clinics program provided infrastructure funding for more than 60 clinics, but did not fund service provision [17]. The program claimed not to prescribe a particular model of service delivery, but key objectives included patient-centred care and effective use of information technology, and key features included co-location of services such as allied health and diagnostic imaging [17]. At the time of the Super Clinic program, Australia was already experiencing a rapid shift in the mode of general practice from small privately owned 2–4 doctor practices to much larger group practices run by health service delivery organisations, with other subcontracted diagnostic services being housed in a single location or building. Multidisciplinary services at the centre in the current study were provided by health professionals with a direct contractual relationship with the medical centre operator as well as more broadly by services with a subcontracting relationship. At the time of the study, the practice consisted of 12 doctors, 6 practice nurses, 11 administrative staff and 9 allied health professionals with a direct contractual relationship with the medical centre operator. An additional 5 services were provided under subcontracting arrangements. Professionals with a direct contractual relationship could access the shared practice management system and patient records, whereas subcontractors managed their own systems and communicated via traditional referral and communication systems such as letter and phone.

Consumers were recruited through the practice nurses, who were asked to identify people with chronic conditions who had seen more than one health professional at the centre. Nineteen consumer interviews were conducted, recruited in two cohorts to participate in different aspects of the overall project. Cohort one (*n* = 10) was recruited for an interview to explore their experiences only. This was a convenience sample recruited by the senior practice nurse and it was observed that this resulted in participants who had seen the same two doctors and a limited range of other health professionals at the centre. Cohort two (*n* = 9) was originally recruited for the patient experience survey testing, but to extend the scope of interview data, were asked to also complete an interview on their experiences. To diversify the sample, consecutive consumers attending a nurse clinic were invited to participate in cohort two. The recruitment for both cohorts was undertaken by practice staff. The number of people who declined to participate was not recorded.

Ten interviews were also conducted with health professional and management staff at the healthcare centre. Staff were invited to participate at a staff meeting attended by the researchers. To provide a range of views in the limited time period available, a purposive sample of GPs, nurses, allied health professionals and managers was subsequently recruited with assistance of the Practice Manager. Sampling was designed to include both male and female GPs and with various lengths of employment at the centre, and we aimed to recruit at least one staff member with experience in the nursing, administrative and allied health aspects of the practice. No participants withdrew from the study before or after completion of an interview.

All interviews were guided by semi-structured protocols: the broad questions are presented in Table 3. The protocols were developed by the research team and reference group to encourage participants to volunteer their own perceptions and understanding of primary health care integration, with prompts related to the dimensions of the typologies [12, 13] used where necessary to more fully explore the existing theoretical basis for the project, including key features such as continuity, information sharing and organisational culture. Due to the time-limited nature of the pilot study, interviews were conducted by three researchers to maximise efficiency in data collection. These interviewers each had qualitative data collection experience, an interest in consumer experience and knowledge of the integration framework (AP, JG and MB). Interviewers discussed the study and the questions beforehand and agreed to adhere closely to the protocols to minimise variations in style during data collection in order to ensure comparability of data. No moderator was present during the interviews. The lead investigator (MB) reviewed audio files for consistency and debriefed interviewers during the during data collection period.

**Table 3:** Broad interview questions

Consumers
1. [This practice] is set up as an integrated primary health care centre. When I say “integrated primary health care”, what sort of things come to mind for you?
2. What are your expectations of [this practice] for care for your chronic condition?
3. Can you tell me about your experiences with [practice] services?
4. We are particularly interested in the effect of integrated health care on the time people spend looking after their health. Can you talk about that?
Providers/managers
1. [This practice] is set up as an integrated primary health care centre. When I say “integrated primary health care”, what sort of things come to mind for you?
2. What are your expectations of working in an integrated primary health care centre?
3. Can you tell me about your experiences with providing services/working at [this practice]?
4. How does [this practice] compare with other general practices where you’ve worked in the past?

Interviews were held in a private consultation room at the healthcare centre: discussions with the reference group identified this as likely to be the most convenient and comfortable location for both consumers and health professionals due to its familiarity and the ability to schedule interviews around appointments. Interviews were digitally audio-recorded and with both groups lasted between five and 25 minutes (average approximately 10 minutes for consumers and 11 minutes for staff). All three interviewers conducted interviews of shorter and longer duration. The interviewers had no relationship or contact with participants prior to the interviews.

### Analysis

The digital audio recordings were transcribed by an external transcribing service and the transcripts reviewed for accuracy by the research team. De-identified transcripts are available in The Australian National University Data Commons [18]. Data were managed using QSR NVivo10 software and were first analysed separately by three members of the research team (MB, TJ and AP). The coding frame presented in Table 4 was finalised by agreement; findings were also discussed with the reference group prior to drafting of publications.

**Table 4:** Summary of major themes, subthemes and dimensions of integration

Theme	Subtheme	Dimensions of integration from typologies
Consumers		
Making meaning from the term integration		-
Experience at the healthcare centre		Person-focused care
	1. Access to services 2. Care continuity 3. Performing above expectations 4. Staff availability and competence 5. Appointments/services running on time 6. Friendly staff 7. Shared attitude	Clinical integration Clinical integration Clinical integration Functional integration Functional integration Functional integration Normative integration
Staff		
Making meaning from the term integration		Person-focused care
Experience at the healthcare centre		-
A cultural shift towards teamwork and care integration		Normative integration Clinical integration Professional/service integration Functional integration

A framework approach [19] combining both independent inductive development of themes and interrogation for the dimensions and characteristics of integration identified in the typologies was used for analysis. Several features of the current study, including the short timeframe, multidisciplinary research team and the need for answers to specific applied questions made the study a good fit with Ritchie and Spencer’s suggested application of framework analysis [19]. As the objective of the study was primarily contextual, concerned with understanding people’s experiences, attitudes and the nature of the system [19], the inductive approach kept a close focus on people’s experiences, ideas and practices, in the context of an integrated health care service. Through an iterative process, experiences were organised into themes generated from the data: as the coding framework developed, earlier transcripts were re-examined to ensure the framework was representative of experiences across interviews [19]. Data were examined for both positive and negative accounts of phenomena. All interviews, regardless of length, were included in the analysis. Data saturation was reached by the twelfth consumer participant in the inductive analysis.

In addition to the inductive analysis, one researcher (MB) also mapped the data against the 21 features and six dimensions of the typologies (see Tables 1 and 2). In the final interpretative phase of analysis, data were organised into broad thematic areas, some with subthemes, based on the inductive analysis and intersections with features of the theoretical typologies identified (Table 4).

Interviews were numbered sequentially within their group (consumer, health professional or manager). Participant quotes used within findings are identified only by their group and number.

## Results

### Consumer experiences

#### Characteristics of participants

Consumer participants in the first interview cohort were not specifically asked for demographic details. All identified as having at least one chronic condition and during the course of the interview, four mentioned that they were retired. Six of the first cohort of consumer participants were female. The second cohort of consumer participants provided demographic details in the patient experience survey completed in addition to the interview. Participants were aged between 54 and 81 years and reported between three and twelve chronic conditions. Six of the participants in cohort two were male, the majority were retired or on disability, had at least some tertiary education and described themselves as financially comfortable.

Table 5 presents the health professionals and services within the health centre building accessed by participants in each cohort. Services offered by providers with a direct contractual relationship with the medical centre operator are marked with an asterisk. Other services were co-located but subcontracted. For the first cohort, services used were explored in the course of the interview and this information extracted and summarised. Participants in the second cohort indicated the services they had accessed in the patient experience survey. Participants in both cohorts were also questioned about their intentions to use other services within the building in future.

**Table 5:** Health services accessed by consumers

Services accessed	Number of consumers *N* = 19
Practice Nurse*	17
Pathology	8
Pharmacy	7
Radiology	5
Dietician*	2
University training physio clinic	2
Specialist*	1
Physiotherapy*	1
Psychology*	1
Diabetes educator*	1
Sleep clinic	1

^*^Services offered by providers with a direct contractual relationship with the medical centre operator are marked with an asterisk

Almost all consumers had a usual GP and regularly saw the practice nurses. They also accessed a range of other services available in the building, particularly pathology and pharmacy. Many commented that they would make greater use of the on-site services such as physiotherapy when the need arose.

### Perceptions, expectations and experiences

Participant experiences of health care, as reported in this study, were overwhelmingly positive. One participant summarised this: “But you know our health system here in Australia is way ahead of America, England, anywhere I've been. So I'm always very happy to be sick here” (Consumer 19).

#### Making meaning from the term integration

Many consumers had not heard the phrase “integrated primary health care”; despite this, all participants were able to describe what they thought the term meant in ways that were consistent with definitions. Given the Super Clinic setting and the publicity that had surrounded this program, a particular focus of perceptions for consumers was that integration was increased through co-location. Consumers frequently used the term “one-stop shop” a phrase that had been used by the Health Minister promoting the original program to illustrate their experiences of integration.

“‘Integrated’ to me indicates that it’s not just a minor health centre where you go and see the GP for minor ailments and things like that but there are other treatments and things available where you can be interviewed for various other things besides just ordinary medical issues, like physiotherapies and things like that. I’ve noticed some of the signs around, you have a chemist available in the building and the X-ray facilities are also integrated into the building as well which makes it a very good medical centre to visit, in my opinion.” (Consumer 01).

“it [integrated primary health care] includes doctors, nurses, so I can get all my shots and my blood and everything done in the one spot, that I can get my X-rays… it’s like a kind of one-stop shop.” (Consumer 02).

#### Experience at the healthcare centre

Several consumer participants reported that they had followed their GP of 20+ years to the healthcare centre because they were satisfied with the quality of care offered by that particular GP and were motivated to seek continuity of care. Whilst not directly examples of integrated care, these comments illustrated the importance of retaining established relationships and the adaptations they were prepared to make.

“Well based on long experience with certain doctors here [laughs] we just mentioned that we’ve known [doctor] for 35 years plus and some of his staff are also here now. I have full confidence in the resources of [healthcare centre]. … the same people and the same good care.” (Consumer 05).

“I want to see the same doctor, because he's had, I don't know, 25, 30 years of contact with me, he's quite a busy man and so instead of being able to see him tomorrow, I might have to wait a few days. But I must say, the girls at the front are very good at fitting you in.” (Consumer 19).

When asked to assess their experiences of the healthcare centre, and to compare with healthcare experiences they had elsewhere, participants overwhelmingly reported satisfaction. Many of the comments reflected **person-focused care** and related to **clinical level** features of integration such as continuity and case management, framed as consumers experience these elements as follows.

##### Access to services

Consistent with their perception of integration as a “one-stop shop,” many participants described the ease of access to multiple services offered by the centre and the concomitant increase in likelihood of them following up on referrals to these services as a result.

“There’s more nurses and that they can fit you in just for a flu shot whenever, or if I needed to have something else done they can fit you in straightaway which is great, and the fact that I can go downstairs and have bloods done if [name] says, you need bloods, and there’s no waiting or expecting for me to go elsewhere, but compared to having to go to [other practice] one time it’s more friendly, everyone’s personable, everyone knows everyone, which is what I like.” (Consumer 02).

“… they said, oh well you need to go to a physio, you should see a physio, well – and this is what happened with the dietician with me, like, I probably honestly would not have got around to ringing up the dietician, to making the appointment, when someone had said – but I walked out the door and said, he wants me to see so-and-so, and she said, I’ll make the appointment for you now.” (Consumer 07).

“… you’ve pretty much got the one stop place where you can come, see your GP, if you need a referral to go and do something you can just go downstairs or up, wherever you need to go and it’s good because for people like me [with multiple sclerosis]. … I think your energy levels get worse and you just don’t have time and the energy to be going to three different places when you could be coming to one and go, I can do everything in the one hit here and it’s done.” (Consumer 10).

However, some participants felt the transport and parking were not ideal. Limited disabled parking and bus schedules that were difficult to follow meant that some people did not find getting to the centre very easy, but they still appreciated having multiple services in one location once they accessed it.

Another participant was concerned at the cost of multiple visits, which they believed should be free since the visits were part of a care plan. This may be interpreted as an example of poor communication between health care practitioners and patient:

“When you’re on the health care plan you’re supposed to have access to certain other medical people like physio or whatever free. It’s not free. … I’ve got appointments with a physio coming up and it’s going to cost me $300 for five visits and then hello here I’m on a pension only, I don’t have other income and that was just, why do they keep saying you get access to these people [for] free. Five visits a year when it’s *not* free, it costs. I can just phone up and make an appointment with a physio and it costs me the same amount.” (Consumer 08).

##### Care continuity

Consumers also expressed confidence in the continuity of care offered by the centre, including the completeness of records. Their comments reflected the importance of having one professional with knowledge of their entire history when dealing with chronic conditions.

“I know that they always send [a] report – even, like, the eye specialist and that who’d been recommended by or referred by here, they all send letters back to the doctor so I know that all the things are going onto my file. Yeah, so she would be my [laughs] primary source of information if anybody needed to know my whole history. I’m fairly confident that most of the stuff that I talk to with the other people actually do all get back to her in the end, yeah.” (Consumer 07).

##### Performing above expectations

**Person-focused care** was reflected in several descriptions of additional efforts by staff to accommodate specific consumer needs, including protecting vulnerable people from risk in the waiting area and avoiding unnecessary consultations for repeat prescriptions.

“… they’ve gone out of their way to make me kind of comfortable. I’ve been going through chemotherapy and when I’ve been here at that time when I’m susceptible to infection I’ve asked about going into – rather than being in an ordinary – this is just an example of the cooperation – rather than being in the waiting room with all the other patients I’ve been allocated my own room there for isolation, to protect me from infection there.” (Consumer 05).

“I forgot to get a prescription repeat for whatever particularly, like, [nurse] and [nurse] and all of those who’ve known me for a long time, they’ll just look up my record and go, oh it’s this one, and they’ll double-check it and go, OK I’ll get [doctor] or [doctor] to write you out a script for that.” (Consumer 10).

Descriptions of **person-focused care** also often included experiences with staff and healthcare organisation consistent with elements of **functional integration**, particularly their experience of service management.

##### Staff availability and competence

Many consumers praised the responsiveness of the administrative staff, who they felt did their best to accommodate consumers’ needs and provide information.

“They’ll give me a choice of times, which is very good … There’s always someone who can answer the phone when you ring to make an appointment or a query, and they’ve always got the information available, which is very good.” (Consumer 01).

“If I walk in, and [receptionist] always knows, or [receptionist] knows…when have you got to come back. Oh gee, that's going to be difficult, but we'll find a spot for you, hang on. And they take the time. And they know when you go to the counter, OK, he's coming in, he needs to see the clinic, or he needs to see the doctor, or he needs… and nothing's a problem.” (Consumer 18).

However, not all experiences with availability were positive. One participant believed that the move to a large centre had made it more difficult to get appointments.

“I ring up one day and I say I want an appointment for the current [day], no, we can't do it. I said, you know, just make an appointment. But you've got… she's this and that, and we have a few spots that we save through the day, and you've got to ring at 7 o'clock in the morning, on the day, and see if we've got… if we can slot you in. Because she only works 'till lunch time, right. And I was on the phone for 20 minutes the other morning before somebody answered. And I thought, well this is just hopeless you know.” (Consumer 15).

##### Appointments/services running on time

Consumers’ opinions were divided on whether the time management at the centre was effective. Some described the centre as efficient, with little evidence of extended waiting times.

“I’m very impressed that everything seems to run on time, it’s efficiently run, which is a bonus, not sitting for hours and waiting. Whether that’ll continue or not I know, hopefully will, but that’s my first impression of – it’s very well run.” (Consumer 08).

“[doctor] is very quick, and likes to be on time, and we like that. We don't like waiting two hours, where we used to at a previous doctor.” (Consumer 14).

Some participants also reported feeling that they had enough time in appointments (which could come at a cost to services running on time):

“I never feel rushed with [doctor] or any of the other doctors. They’re prepared to listen to either [name] or I with our medical problems and advise us what to do.” (Consumer 09).

“… she’ll exhaust every avenue until the appointment’s finished, so no I never feel – and even when I’ve seen [doctor] – never feel rushed, just do what I’ve got to do and that’s that, so it’s good.” (Consumer 10).

However, not all participants shared these feelings. Some reported feeling rushed and guilty about the amount of time they took with the doctor.

“I know that whenever I get out and I go to make my next appointment the receptionist staff is constantly asking, do you need a double appointment, do you need a double appointment, and it’s all, no I don’t. Like, it’s done, and that kind of makes me feel like, oh have I been in there too long, have I – do you know what I mean? It makes me feel like I’ve done something wrong. Yeah, that makes me feel a bit – that makes me feel more rushed, actually being in with the doctor.” (Consumer 06).

##### Friendly staff

Participants reported that they appreciated feeling known by staff and they perceived staff within the centre as friendly. This included the GPs, receptionists, nurses, pharmacists and even the café personnel. The café personnel were considered by some to be equally important to their general feeling of ‘being known’;

“Respondent: … now they’ve got the chemist downstairs, and a coffee shop …, so I think you’re pretty much covered in most of your…

Interviewer: Cover your caffeine as well as your meds [laughs].

Respondent: Yeah especially depending on the appointment yeah [laughs].

Interviewer: They make a good coffee, too, I had a couple this morning [laughs].

Respondent: Oh they’re fabulous and the guy there’s really lovely, I like him, so he’s good value, so…

Interviewer: Yeah seems like a nice guy.

Respondent: Yeah that makes it nice too especially if you’re feeling a bit stressed or something, you just need a friendly face to have a bit of a laugh.” (Consumer 10).

##### Shared attitude

Consumer participants had a sense of the collective attitude, trust and collaboration indicative of **normative integration**. One participant commented that this difference might even be overwhelming for new people, suggesting they felt it to be a strength of the healthcare centre.

“I think someone new coming in would find the experience almost overwhelming, it's all here, and if they actually get the care and treatment that makes it even better. And I think it's important they're all on that… the wavelength of wanting to help people.” (Consumer 18).

Participants reported that they were so satisfied with the health service that they recommended it to family members and friends.

“I’ve certainly brought my mother – like, my mother had to have some imaging things done and she’s going … cause [other clinic] is such a pain to, you know, go to as well. There’s one at [healthcare centre] so I’ve made sure she came here and things like – because of the convenient parking and the, you know, cause it’s closer proximity and yeah. Mainly the parking has a lot to do with that.” (Consumer 07).

## Staff experiences

Ten staff participated in interviews: four GPs, one practice nurse, two managers, and three allied health professionals. To maintain confidentiality, no demographic details were recorded for the staff participants.

### Making meaning from the term integration

When asked to describe integration, staff participants described it in terms of diverse services being co-located, patient-centric, and optimising continuity of care. They also described integration in terms of care providers having ready access to other care providers in order to seek advice or manage individual case needs (**person-focused care**). Staff participants said:

“Integrated primary health care should be where there’s good co-ordination between allied health and medical practitioners and nursing staff, working towards the better health of patients.” (Health professional 01).

“… communication, liaison, the ability to not just send emails, and it’s to actually to be able to verbally communicate via telephone, or even walking into each other’s rooms. And that’s… what I see from that, patient coming in with a complaint or a condition, it’s not just handled by one person, and then have to either (a) not contact the other person at all and just feed through the patient, but rather the patient can turn up to the clinic and go, “Does the physiotherapist know all my background,” and the idea is, yes, because all the information that we store between the physio, the doctor, the podiatrist, and even the dietician, is all stored on our system, and therefore we’ve got a good rounding knowledge about what it is that this patient’s complaint is, and we can also liaise quite easily on our same system, and through other physical means, of how we can interact around their care.” (Health professional 02)

### Experiences at the healthcare centre

Some staff members had specifically chosen to work at the healthcare centre because it was a GP Super Clinic. Despite this, both the health professionals and managers interviewed did not offer much information on what they expected of this type of centre. In addition to shared information and a high standard of care, one staff member suggested that they expected improved working conditions, such as backups/locums for doctors. Other staff members expected to provide more holistic care in an integrated centre, but suggested this may come with a need for wider knowledge.

“Just you know broader range of knowledge. I’ve learnt a lot since I’ve been here about the different you know areas of health and things like that.” (Manager 01).

“… that’s a very important part of I think integrated health, that you’ve got a team of people that the patients feel connected with, that they can ask for advice from, and get different levels of expertise and in different areas.” (Health professional 01).

One staff participant described their motivation to join the healthcare centre in terms of the needs of patients not being met in a small general practice. By moving, they said, “We’ve lost the intimacy of a little private practice, but there’s been so much more to gain” (Health professional 03).

#### A cultural shift towards teamwork and care integration

Staff experiences provided a strong sense of the collective attitude and collaboration underpinning **normative integration** as an enabler of integrated care. Elements of **clinical**, **professional** and **functional integration** were all clearly identifiable contributors to a culture of integration as reported by GPs, allied health professionals and managers alike.

A sense of teamwork and holistic care was reported as being an important element in the healthcare centre, one that staff participants had either not experienced in previous settings or had experienced but to a lesser degree.

“Yeah, definitely the communication channel is a lot more open. Not only the… can you knock on the door, or go and talk to another health professional directly, but we have on the computer system all the patient notes available, we can send… you know request to have meetings with other health professionals to set up more… more flexibility I guess with catching up with people … I think the whole philosophy here is so embraced by all the staff members. Everyone is so helpful and welcoming. I think it’s the culture of what [the practice] is trying to promote really does resonate with everyone that works here. I think it’s a very supportive environment not only for the clients, but also for the staff members.” (Health professional 04).

Staff participants described a desire to see a real cultural shift in the sharing of information between practitioners of different modalities. They saw the healthcare centre as being a trailblazer towards this shift.

“I think GPs and Allied Health sometimes struggle with the concept, and they need to just be open to the thoughts of their files being shared, their clinical notes being shared. Generally a GP initially shares clinical notes with … their colleagues, not with external providers such as Allied Health, so I think the knowledge basis of what integration can do, can be improved.” (Manager 02).

Key to the success of such cultural shifting, they explained, was communication. Formal modes of communication, such as writing clear and structured patient notes and sharing them, were described. Of equal value to the cultural shift was informal communication and developing a sense of friendship and community with staff of different modalities using the space.

“Communication, it has to be strong, and it’s time consuming. And obviously communication can be taken, especially written communication, can be taken in many ways, so just being careful of how things are communicated, to what extent, what detail.” (Manager 02).

“I found that obviously as physios we have our own acronyms, and I think GPs have their own acronyms, so I think it’s something that I try to portray to the other physios here too, because this is the first time they’ve worked in this environment. If they write TVA, or if they write FIS, … FIS for a doctor could mean FIS differently to what we think. So we’re starting to write flexion standing, rather than FIS.” (Health professional 05).

One staff participant explained that although the ideology of sharing information between practitioners to optimise patient care was generally held by staff at the healthcare centre, and that it was facilitated by the shared electronic information system, that difficulties arose in relation to sharing mental health information, which was partially addressed by psychologists providing notes that staff could read that were different from the patient notes.

“… someone having counselling, that they may disclose something that they would never have told me, you know, or… yeah. And that’s a difficulty of how much. And I’m not sure with the psychologist here how much… I think she keeps a lot of her own records, so I’ve never looked to see. But I know the physio does put records in, and I know he looks at what we’ve got as well, too, to help him, and I’m the same. So they can look, and for instance the dietician can look and see what the latest cholesterol was, and you know things like that that are very useful to be… so it’s just when you get that sort of super confidential bit of information, and that’s probably more policies and procedures. … they will give us a summary, or you know when there’s times that we’ve got to perhaps do a referral to a psychiatrist, and give us some information there. But they actually keep totally separate notes.” (Health professional 01).

Also key to the cultural shift toward integration, was the way space was used. Room sharing and communal areas were described as facilitating communication and care integration.

“I love the communication here. I love the fact that like in our section there, we’re all… like the room that we’re in right now it’s an Allied Health room, which means everyone’s to work as a team. It’s not like the physiotherapist’s room, or like the podiatrist’s room, it’s the Allied Health room, so it’s got a really strong outlook in that getting everyone on board.” (Health professional 05).

“[we do] A lot of horse trading in the corridors and tearooms. … it’s quite… a bit of a different flavour to what people are willing to type as opposed to as what they’re willing to say” (Health professional 06).

When describing the value of the cultural shift, one participant summed it up in the following way: “I’ve seen the lot, as in solo practice with no nurses, no practice manager, and I wouldn’t go back to that system by choice again.” (Health professional 06).

## Discussion

The healthcare centre was still relatively new and growing, but the interviews provided evidence that many dimensions of integration were already becoming established and consumers were experiencing integrated care. Consumers’ experience of integrated care tended to be implicit in their descriptions of primary health care experiences more broadly. Their comments that could be related to the typologies [13, 14] were primarily about clinical and functional integration, particularly their experiences of continuity of providers, the usefulness of the shared information systems and the helpfulness of front desk staff. Staff had a strong focus on clinical level integration, but also talked about a cultural shift that demonstrated normative, professional and functional integration. Across all groups, discussion of normative integration was primarily in terms of a sense of collective attitude. There was very little discussion of organisational or system level integration.

Some specific issues arose that may be important to investigate in the implementation of integrated primary healthcare more broadly. Consumers and staff both liked the ease of communication and continuity provided by the shared record system. Consumers liked the idea that their entire medical history and notes from other providers may be in one place and available to their GP and other treating professionals in the centre. In contrast, some allied health professionals suggested that sensitive information should not necessarily be available to all providers accessing the system. This suggests shared records may have an important part to play in facilitating integrated care, although careful consideration may need to be given to restricting access to potentially sensitive details in consultation with the preference of the consumer. The availability of effective and compatible computer technology to facilitate these functions is also important for informing the extent to which information is shared electronically for integrated care [20–22].

Work specifically investigating the role of information systems in continuity and integration, both in the current study and previous research, has found that factors such as inconsistent record-keeping and terminology undermine their utility [16, 23]. It is therefore not surprising that both consumers and providers spoke extensively about the value of the familiarity between professionals enabled by co-location as an important feature of the healthcare centre. Interaction with people from other services, often in the staff tearoom, helped to establish relationships and build trust which then translated to comfort with recommending these services to consumers. Medical and allied health professional staff enjoyed the informal consultations with each other and the ability to “stop by” and discuss things without the need for formal referral or response letters when they were not appropriate. Consumers spoke of the convenience of having services all in one building, many able to be booked through the same reception staff as their GP. Consumers’ descriptions conveyed a confidence in the services associated with their GP, especially where they had a long-standing relationship that pre-dated the creation of the Super Clinic.

Overall, this meant that not only was there evidence of the more “practical” elements of integration such as information flows, there was evidence of the development of a shared culture, noticeable to the consumers as well as staff. Rather than a focus on more business-like concepts such as coherence of polices and management, integration was described in terms of the way people interacted with one another and the ease of navigating healthcare. The value of relationships and the influence of co-location on their development and maintenance will be an important focus of broader work on models of integrated primary healthcare.

An investigation of overall coding patterns across all consumer and staff interviews against Fulop et al’s [12] typology and Valentijn et al’s [13, 14] key features suggested that these frameworks may be useful for capturing the way people in service provision, especially health professionals, describe integration, but less useful for consumers’ experience of integrated care. Staff described a sense of collective attitude indicative of normative integration, underpinned by examples of clinical, professional and functional integration such as holistic care, improved communication and teamwork. Their responses tended to be framed similarly to the descriptors included in the Valentijn et al. [13] taxonomy, which was developed from a professional perspective. Clinical and functional dimensions were evident in the way consumers described their experiences of care such as continuity of providers, the usefulness of the shared information and the importance of administrative staff. They also reported a sense of collaboration characteristic of normative integration. However, dimensions that operate at the meso and macro level (professional, organisational and systemic integration) were absent from consumers’ data and much of the way they framed their experiences fell outside the framework descriptors, focusing instead on elements of quality health care more broadly, such as access. Valentijn et al. [13] acknowledged that their Delphi process did not involve some key stakeholders, including consumers, suggesting work in local settings may further refine understanding of key features across groups. The current study provides evidence that work is still needed on the key concepts of quality integrated primary health care from the consumer perspective. A potential area of focus may be the intersection of integrated primary health care with dimensions of consumer experiences of health care more broadly [24].

### Limitations

As a study pilot, there are a number of limitations to acknowledge. The study was conducted at one healthcare centre, constructed and promoted as part of a government program, the GP Super Clinic Program. It is not known whether findings would generalise to other GP Super Clinics or to other types of integrated primary healthcare centres. Consumer participants were also recruited by practice nurses, with interviews for cohort one arranged and scheduled by one nurse with whom many consumers had a long-term relationship. This may have biased the findings on the importance of continuity. Interviews were conducted by three researchers which may have affected the consistency of questioning and rapport with participants. The short duration of interviews and the small numbers of participants may also have affected data saturation and representativeness, particularly for health professionals. Finally, demographic information was not collected for most participants, limiting the conclusions that could be drawn regarding diversity of the sample.

## Conclusion

Existing frameworks for integration have been heavily influenced by the provider and organisational perspectives. They are useful for conceptualising integration from a professional perspective, but are less relevant for consumers’ experiences. Consumers of integrated primary health care may be more focussed on relational aspects of care and outcomes of the care with less focus on the organisational or structural processes necessary to produce them. Despite this, as proposed by previous authors [3, 12–14], the feature that unifies experiences of both providing and receiving integrated care is a sense of collective attitude. Thus, consistent with Kodner and colleagues’ [1] distinction between integration as a process and integrated care as an outcome, future research into these concepts should focus on the area salient to each group and the influence of a shared culture for both.

## Abbreviations

GP: General practitioner
